# Redescription and phylogenetic analysis of the type species of the genus *Panagrellus* Thorne, 1938 (Rhabditida, Panagrolaimidae), *P. pycnus* Thorne, 1938, including the first SEM study

**DOI:** 10.21307/jofnem-2021-080

**Published:** 2021-10-01

**Authors:** Joaquín Abolafia, Matteo Vecchi

**Affiliations:** 1 Departamento de Biología Animal, Biología Vegetal y Ecología, Universidad de Jaén, Campus “Las Lagunillas” s/n. 23071 Jaén, Spain; 2 Department of Biological and Environmental Science, University of Jyvaskyla, PO Box 35, FI-40014, Jyvaskyla, Finland

**Keywords:** 18S rDNA, 28S rDNA, Description, Molecular analysis, Morphology, Phylogeny, SEM, Tarantobelinae n. subfam., Taxonomy

## Abstract

The identity of *Panagrellus pycnus*, the type species of the genus *Panagrellus*, is discussed after studying specimens from a cultured population collected in Italy that fits the original material of the species. A new characterization is consequently provided as follows: body 0.93–1.32 mm long, lip region continuous with the adjoining body, stoma with gymnostom very reduced, pharynx with not swollen metacorpus, neck 161–203 µm long, excretory pore at level of the metacorpus, post-vulval uterine sac 99–162 µm long or 2.6–3.8 times as long as the body diameter divided in a short tubular proximal part and a long swollen distal part, vulva post-equatorial (*V* = 63–69), female tail conical elongate with acute terminus (133–170 µm, *c* = 6.8–8.1, *c’* = 4.9–7.0), male tail conical elongate with acute terminus (104–137 µm, *c* = 7.8–10.9, *c’* = 3.6–5.1), and spicules 70–81 µm long having angular hook-like and very curved ventrad lamina ending in a spatulate tip with a refringent forked axis. The evolutionary relationships of this species and the genus *Panagrellus*, as derived from the analyses of 18S and 28S rDNA fragments, are discussed. Additionally, the phylogenetic relationships among the members of the infraorder Panagrolaimomorpha is studied, being the genus *Tarantobelus* transferred to the family Panagrolaimidae and the new subfamily Tarantobelinae n. subfam. is proposed to accommodate it.

*Panagrellus* Thorne, 1938 is an infrequent genus belonging to the family Panagrolaimidae Thorne, 1937 (proposed as subfamily by Thorne (1937) and erected to family by Paramonov (1956)), and includes 15 species (Abolafia et al., 2016; Ivanova et al., 2018). This genus is characterized by having usually ventral curved spicules with hooked manubrium and bifurcate lamina tip. Its type species, *P. pycnus*, was described and succinctly illustrated by Thorne (1938) on the base of male and female specimens collected in slime secreted by wounds from the trunk of Great Plains cottonwood tree in Utah, USA. Later, Goodey (1943) transferred this species to the new erected genus *Turbator* Goodey, 1943 (currently junior synonym of *Panagrellus* together to *Tylorhabdus* Sukul, 1971). Andrássy (1958, 2005) and Varga (1958) reported this species from organic matter undergoing fermentation in Hungary. Hechler (1971a) revised the Thorne’s material and redescribed *P. pycnus* providing some line illustrations of the stoma and of the male and female posterior ends. This species is distinguished from its congeners by having spicules with angular hook-shaped manubrium and lamina very thin lacking dorsal hump and bearing spatulate terminus having forked refringent axis.

Currently, only five species of the genus [*P. ceylonensis* Hechler, 1971b, *P. dubius* Sanwal, 1960, *P. levitatus*, Ivanova, Perfilieva and Spiridonov, 2018, *P. redivivoides* Goodey, 1943 and *P. redivivus* (Linné, 1767) Goodey, 1945] have been characterized molecularly and used for phylogenetic analyses.

In the present paper, a cultured population of *P. pycnus* collected in Italy and used as food for carnivorous tardigrades is studied providing new LM and SEM illustrations and molecular data.

## Materials and methods

### Nematode extraction and culture

The nematodes were initially obtained from rotting pears on the ground in Borgata Città, Bologna, Italy (44°34′42.7″N 11°10′26.6″E; 27th October 2019; leg. Matteo Vecchi). Other than nematodes, the fruits were heavily colonized by *Drosophila* spp. and Nitidulidae beetles. Fruit pulp was spread on Yeast-Sucrose Agar (Yeast extract 1%, Sucrose 2%, Agar 1%) plates and after 1 week of incubation at 21°C a single gravid female was handpicked with a loop to start an isofemale line. Nematodes were mass cultured at 21°C in 0.3 L plastic containers on a substrate composed of 19 g of whole-grain wheat breakfast cereal (Weetabix®), 3 g of dry brewer’s yeast and 60 mL of distilled water.

### Nematode processing

The specimens were killed by heating, fixed in a 70% ethanol solution, transferred to pure glycerin following the Siddiqi’s (1964) technique, and mounted on glass microscope slides with the glycerine-paraffin method (de Maeseneer and d’Herde, 1963) somewhat modified using hot liquid paraffin.

### Light microscopy (LM)

Observations were made and measurements were taken using a Nikon Eclipse 80i (Nikon, Tokyo, Japan) microscope with a drawing tube (*camera lucida*) attached to it. Demanian indices and other ratios were calculated according to de Man (1881). Pictures were taken with a Nikon microscope equipped with differential interference contrast (DIC) optics and an associated Nikon Digital Sight DS-U1 camera. Micrographs were combined using Adobe® Photoshop® CS. The terminology used for the morphology of stoma and spicules follows De Ley et al. (1995) and Abolafia and Peña-Santiago (2017), respectively.

### Scanning electron microscopy (SEM)

Specimens preserved in glycerine were selected and prepared for observation under SEM according to Abolafia (2015). They were cleaned in distilled water, dehydrated in a graded ethanol-acetone series, critical point dried, coated with gold, and observed with a Zeiss Merlin microscope (5 kV) (Zeiss, Oberkochen, Germany).

### Molecular analyses

#### DNA extraction, PCR, and sequencing

Nematode DNA was extracted from single individuals using a modified DNA extraction and PCR assays described by Castillo et al. (2003) and Archidona-Yuste et al. (2016). The specimens were cut in small pieces using a sterilized dental needle on a clean slide with 18 ml of TE buffer (10 mM Tris-Cl + 0.5 mM EDTA; pH 9.0), transferred to a microtube and adding 2 μ l proteinase K (700 μ g/ml^‒1^) (Roche, Basel, Switzerland), and stored to –80°C within 15 min (for several days) until processing. The microtubes were incubated at 65°C (1 hr), then at 95°C (15 min). For DNA amplification, 3 μ l of the extracted DNA was transferred to a microtube containing: 0.6 μ l of each primer (10 mM), 3 μ l Master Mix Taq DNA Polymerase (5x Hot FirePol Blend Master Mix) and ddH2O to a final volume of 20 μ l. The primers used for amplification of the region of 18S rRNA gene were the forward primer SSU F_04 (5′-GCTTGTCTCCAAAGATTAAGCC-3′) and the reverse primer SSU R_26 (5′-CATTCTTGGCAAATGCTTTCG-3′) (Blaxter et al., 1998). The primers used for amplification of the D2-D3 region of 28S rRNA gene were the forward primer D2A (5′-ACAAGTACCGTGAGGGAAAGTTG-3′) and the reverse primer D3B (5′-TCGGAAGGAACCAGCTACTA-3′) (De Ley et al., 1999; Nunn, 1992). PCR cycle conditions were as follows: one cycle of 94°C for 15 min, followed by 35 cycles of 94°C for 45 sec + annealing temperature of 55°C for 45 sec + 72°C for 45 sec, and finally one cycle of 72°C for 5 min. After DNA amplification, 5 μ l of product was loaded on a 1% agarose gel in 0.5% Tris-acetate-EDTA (40 mM Tris, 20 mM glacial acetic acid and 2 mM EDTA; pH = 8) to verify the amplification using an electrophoresis system (Labnet Gel XL Ultra V–2, Progen Scientific, London, UK). The bands were stained with 1.25 µl RedSafe (20,000x) previously added to the agarose gel solution (25 ml). The sequencing reactions of the PCR products were performed at Sistemas Genómicos (Paterna, Valencia, Spain) according the Sanger et al. (1977) method. The DNA sequences obtained for *P. pycnus* (MZ656001 for the 18S rDNA and MZ656000 for the 28S rDNA) and *Tarantobelus arachnicida* Abolafia and Peña-Santiago, 2018 (MZ655998–MZ655999 for the 18S rDNA and MZ656002–MZ656003 for the 28S rDNA) were submitted to the GenBank database.

#### Phylogenetic analyses

For phylogenetic relationships, the analyses were based on 18S and 28S rDNA fragments. The newly obtained sequences were manually edited using BioEdit 7.2.6 (Hall, 1999) and aligned with other 18S and 28S rRNA gene sequences representative of Panagrolaimomorpha and closely related taxa available in GenBank (accession numbers available in Supplementary Table 1) with MAFFT ver. 7 (Katoh and Toh, 2008; Katoh et al., 2002) with the G-INS-i method (thread = 4, threadtb = 5, threadit = 0, reorder, adjustdirection, anysymbol, maxiterate = 1,000, retree 1, globalpair input). Alignments ends were trimmed using MEGA7 (Kumar et al., 2016) up to 1,851 and 1,195 bp for 18S and 28S respectively. The best-fit models of nucleotide substitution used for the phylogenetic analysis were selected using jModelTest 2.1.10 (Darriba et al., 2012). Sequences were concatenated with the R package ‘concatipede’ v1.0.0 (Vecchi and Bruneaux, 2021). The phylogenetic tree was generated with Bayesian inference method using MrBayes 3.2.6 (Ronquist et al., 2012). *Plectus aquatilis* was used as outgroup for the Rhabditida phylogenetic tree. The phylogenetic analyses were initiated with a random starting tree and run with the Markov Chain Monte Carlo (MCMC) (Larget and Simon, 1999) for 2 x 10^7^ generations. The posterior tree distributions trace plots and ESS were checked with Tracer (Rambaut et al., 2018). The tree was visualized and saved with FigTree 1.4.4 (Rambaut, 2018).

## Results

**Figure 1:**
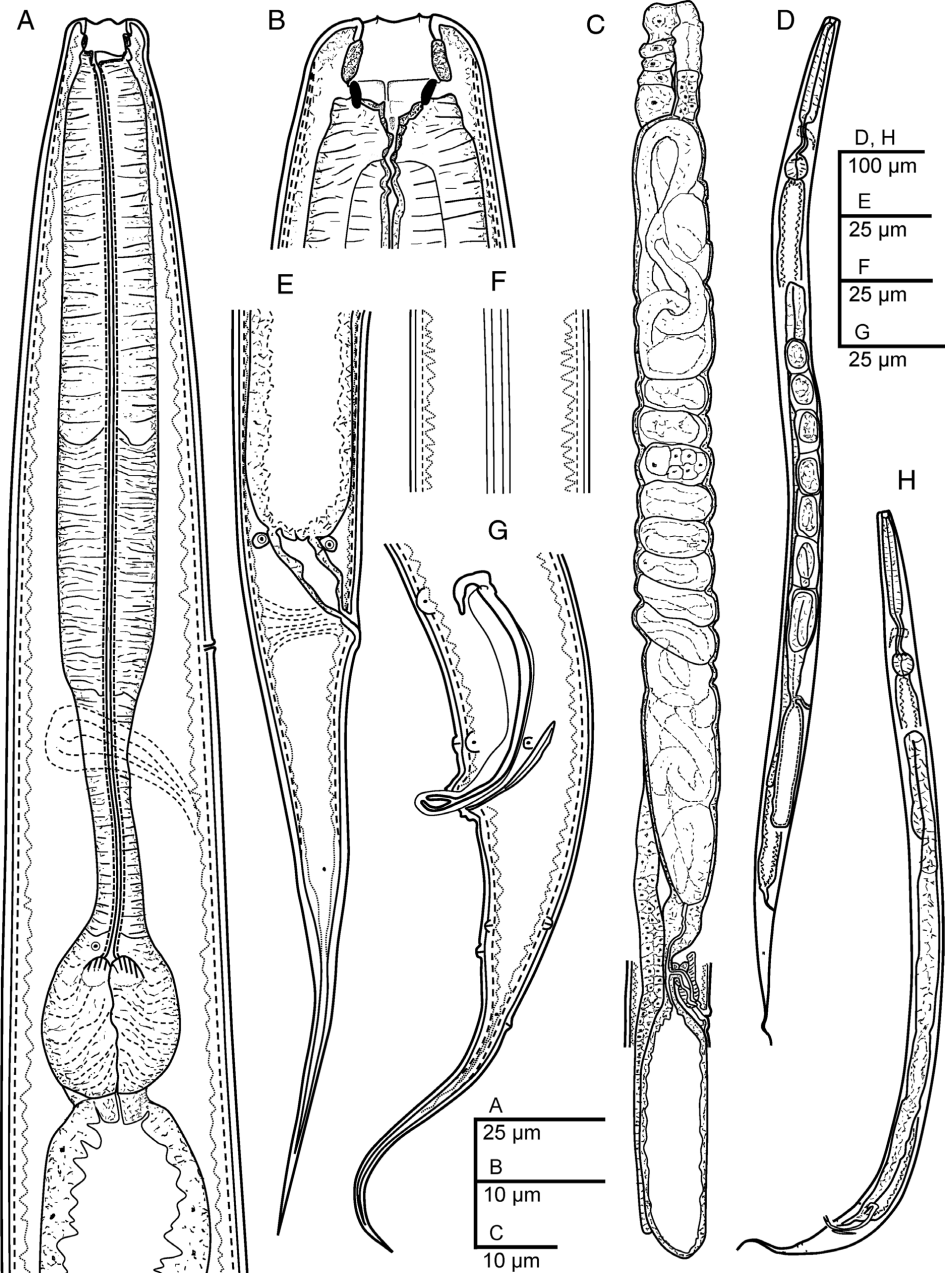
*Panagrellus pycnus* Thorne, 1938 (line drawing). (A) Neck; (B) Anterior end; (C) Female reproductive system; (D) Entire female; (E) Female posterior end; (F) Lateral field; (G) Male posterior end; (H) Entire male.

**Figure 2:**
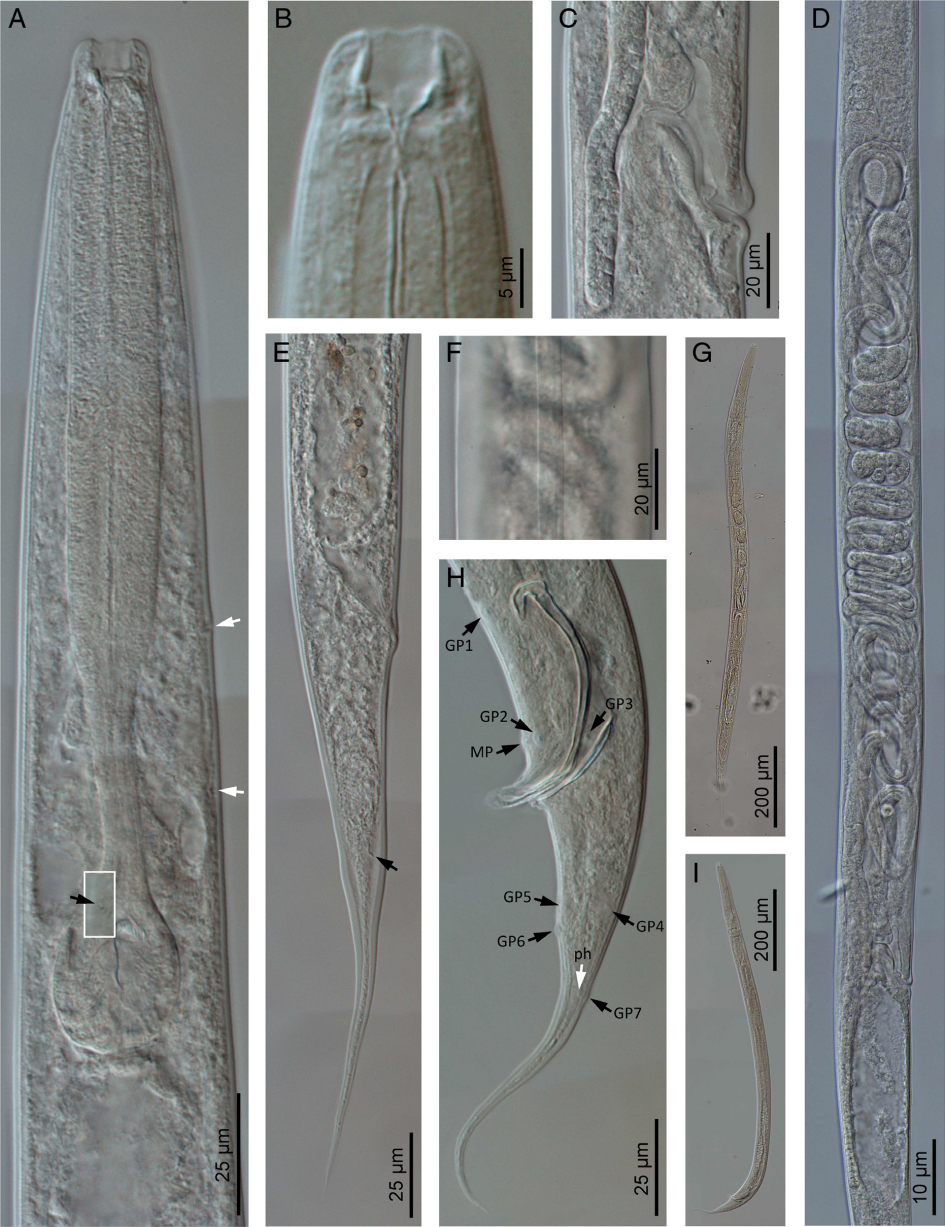
*Panagrellus pycnus* Thorne, 1938 (light microscopy). (A) Neck (anterior white arrow pointing the excretory pore, posterior white arrow pointing the hemizonid, black arrow pointing the deirid); (B) Anterior end; (C) Vagina; (D) Female reproductive system; (E) Female posterior end (arrow pointing the phasmid); (F) Lateral field; (G) Entire female; (H) Male posterior end (black arrows pointing genital papillae, GP; mid-ventral papillae, MP; white arrow pointing the phasmid, ph); (I) Entire male.

**Figure 3:**
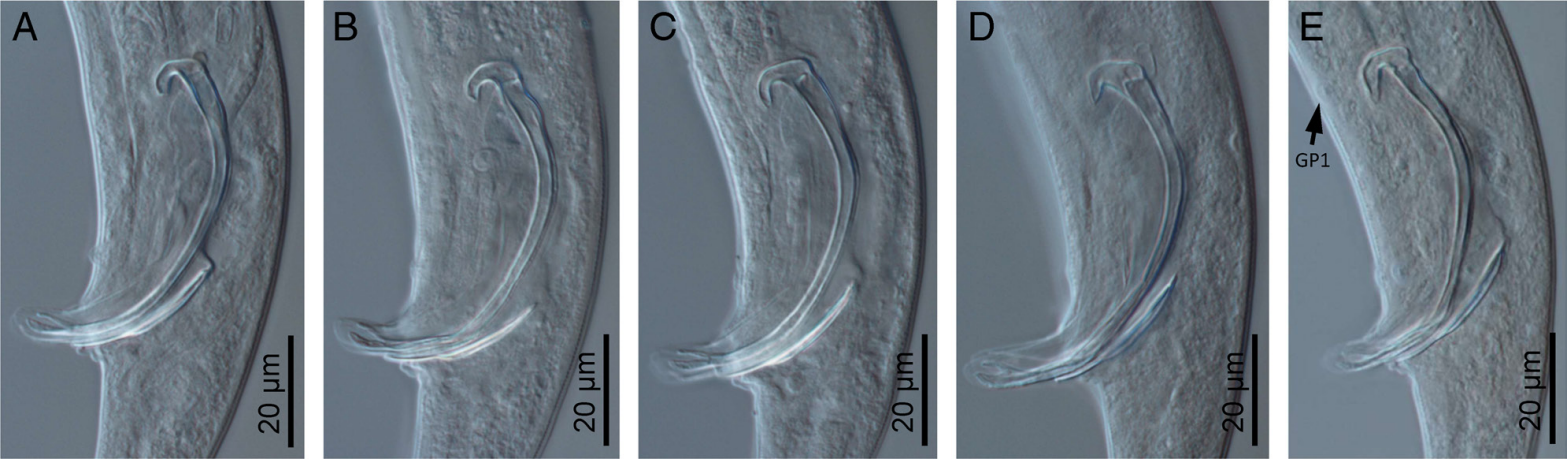
*Panagrellus pycnus* Thorne, 1938 (light microscopy). (A–E) Morphological variability of spicules and gubernaculum.

**Figure 4:**
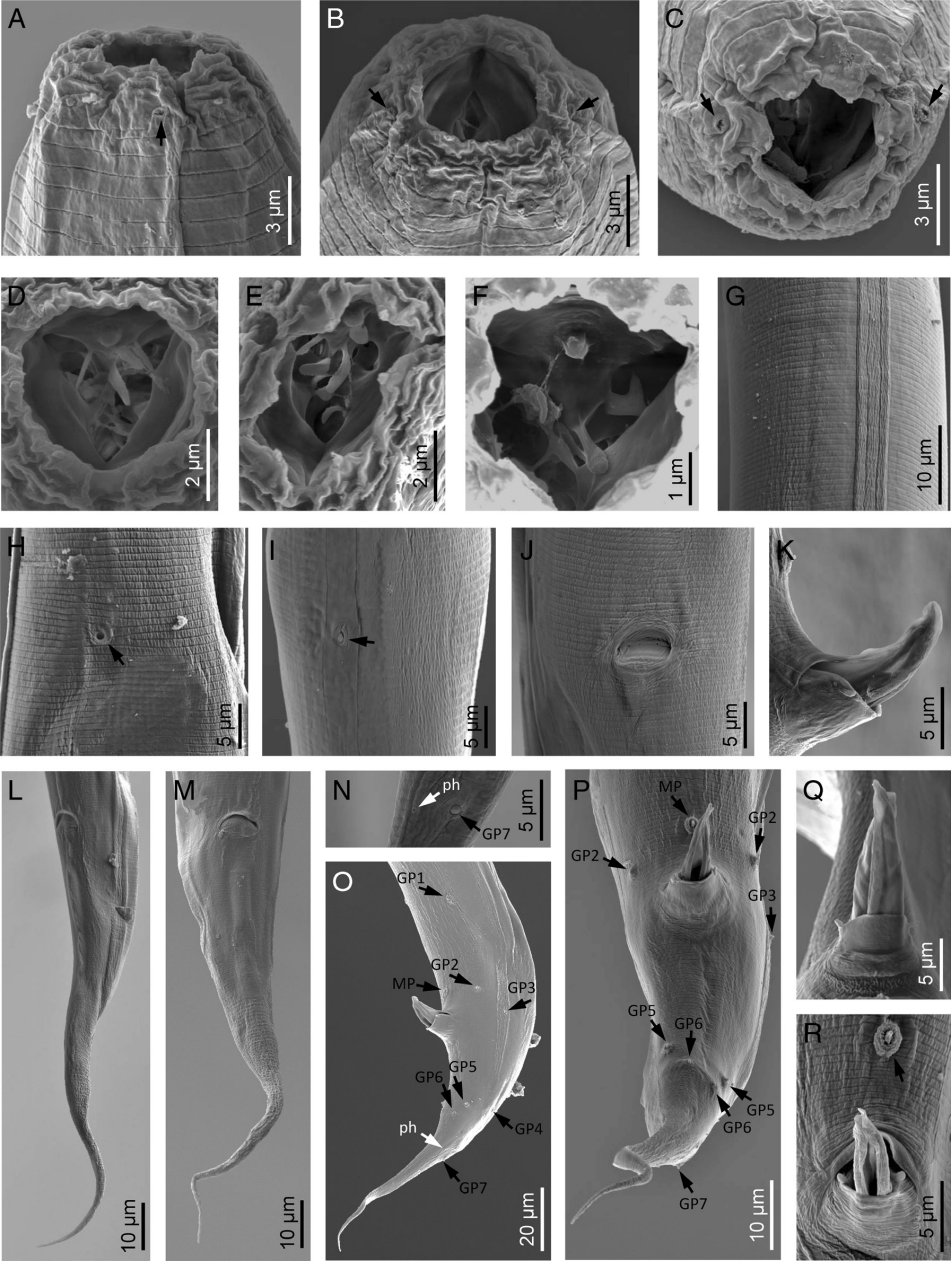
*Panagrellus pycnus* Thorne, 1938 (scanning electron microscopy). A–C: Lip region in lateral, ventral and frontal views, respectively (arrows pointing the amphids); D–F: Stomatal denticles; G: Lateral field; H: Excretory pore; I: Deirid; J: Vagina; K, Q: Spicules tip in lateral and dorsal views, respectively; L, M: Female posterior end in lateral and ventral views, respectively; N: Male phasmid; O, P: Male posterior end in lateral and ventral views, respectively (black arrows pointing the genital papillae, white arrow pointing the phasmid); R: Mid-ventral papillae (arrow).

*Panagrellus pycnus* Thorne, 1938 (Figs. 1–4)

= *Turbator pycnus* (Thorne, 1938) Goodey, 1943

Material examined: 10 females and 10 males obtained from culture.

### Measurements

Measurements are provided in Table 1.

**Table 1.** Morphometrics of *Panagrellus pycnus* Thorne, 1938 obtained from culture.

Sex	Female	Male
n	10	10
Body length (*L*)	1,114.2 ± 103.5 (993–1258)	1,022.0 ± 117.5 (931–1,322)
a	26 ± 2.0 (23.6–29.6)	27 ± 1.6 (24.6–29.9)
b	6.9 ± 3.4 (5.3–16.5)	5.4 ± 0.5 (4.6–6.5)
c	7.5 ± 0.4 (6.8–8.1)	8.6 ± 0.9 (7.8–10.9)
c'	6.0 ± 0.6 (4.9–7.0)	4.4 ± 0.5 (3.6–5.1)
V	66.5 ± 1.8 (63–69)	–
Lip region width	14.8 ± 1.2 (12–16)	14.1 ± 0.7 (13–15)
Stoma length	13.8 ± 1.4 (12–16)	12.7 ± 1.2 (10–14)
Pharyngeal corpus length	109.4 ± 4.9 (104–120)	107.4 ± 6.8 (93–114)
Isthmus length	39.6 ± 5.7 (30–50)	40.0 ± 5.7 (32–53)
Bulbus length	27.9 ± 2.0 (24–31)	27.8 ± 2.5 (24–33)
Pharynx length	176.1 ± 6.6 (167–190)	175.2 ± 11.8 (151–189)
Nerve ring – anterior end	148.3 ± 8.8 (137–160)	139.7 ± 11.0 (122–161)
Excretory pore – anterior end	128.9 ± 7.5 (120–139)	115.8 ± 11.0 (104–139)
Deirid-anterior end	153.3 ± 9.9 (142–160)	163.0 ± 6.1 (156–167)
Neck length	189.9 ± 6.8 (181–203)	187.9 ± 12.5 (161–202)
Body diameter at neck base	37.9 ± 3.1 (34–44)	35.1 ± 2.7 (33–42)
Body diameter at midbody	42.4 ± 4.6 (37–52)	37.6 ± 4.1 (32–47)
Lateral field width	5.7 ± 2.1 (4–8)	6.3 ± 1.0 (5–8)
Anterior ovary or testis length	551.4 ± 49.5 (463–650)	233.0 ± 41.7 (188–289)
Anterior oviduct length	56.1 ± 15.8 (40–91)	–
Anterior uterus length	377.4 ± 63.8 (287–496)	–
Post-vulval uterine sac length	133.4 ± 22.2 (99–162)	–
Vagina length	42.0 ± 8.5 (36–48)	–
Vulva – anterior end	740.9 ± 74.0 (668–863)	–
Rectum or cloaca length	28.2 ± 3.8 (20–32)	9.8 ± 0.7 (9–11)
Anal body diameter	25.1 ± 2.8 (22–30)	27.2 ± 3.4 (23–34)
Tail length	148.9 ± 11.9 (133–170)	119.6 ± 10.1 (104–137)
Phasmid - anus distance	55.6 ± 6.1 (46–67)	47.7 ± 6.7 (36–57)
Spicules length (arc)	–	77.4 ± 3.3 (70–81)
Gubernaculum length	–	30.0 ± 1.2 (29–32)

**Notes:** Measurements in μ m and in the form: mean ± standard deviation (range) where appropriate. Demanian indices (de Man, 1881): *a* = body length/body diameter; *b* = body length/pharynx length; *c* = body length/tail length; *c’* = tail length/anal body diameter; *V* = (distance from anterior region to vulva/body length)x100.

### Description

#### Adult

Moderately slender to slender nematodes of small size, 0.93–1.32 mm long. Upon fixation, habitus nearly straight in females or somewhat curved ventral, J-shaped, in males. Cuticle 1–2 µm thick, bearing minute transverse striations, with annuli 1–2 µm wide. Lateral fields occupying 10–20% of mid-body diameter, with four longitudinal incisures or three alae. Lip region continuous with the adjacent body: lips six, separate, slightly rounded, with protruding, rounded labial and cephalic sensilla; primary and secondary axils with similar morphology, primary ones slightly deeper. Oral opening large, with smooth margin. Amphids very small, oval, located at middle length of lateral lips. Stoma panagrolaimoid, 0.7–1.1 times the lip region diameter long: cheilostom large, slightly wider than long, with strongly refringent, bar-shaped rhabdia, posteriorly thicker; gymnostom very reduced, with small rhabdia; stegostom funnel-shaped with poorly refringent rhabdia, metastegostom bearing dorsal acute rhabdia (dorsal tooth). Pharynx also panagrolaimoid: pharyngeal corpus robust, subcylindrical, 2.2–4.0 times the isthmus length, with procorpus and metacorpus not well discernible; isthmus comparatively thin; basal bulb ovoid, with both valvular apparatus and posterior haustrulum well developed. Cardia small, surrounded by intestinal tissue. Nerve ring at 70–85% of neck length from the anterior end, surrounding the anterior part of isthmus. Excretory pore at 52–75% of neck length, at level of the posterior part of metacorpus. Hemizonid located at level of isthmus. Deirids 78–90% of neck length, at level of isthmus-bulb junction. Intestine without distinct specializations, but with slightly thinner walls at cardiac part; intestinal lumen with rest of diatom frustules.

#### Female

Reproductive system monodelphic-prodelphic. Ovary very long, lacking flexure at post-vulval region, having oocytes arranged in several rows at its distal part and then in only one rows at its proximal part. Oviduct short, slightly longer than the body diameter developing a scarcely discernible spermatheca at its proximal part. Uterus very long, 6.8–10.9 times as long as body diameter, tubular, frequently including uterine eggs (20–33 × 28–54 µm) inside in different stages of development. Post-vulval uterine sac well developed, 2.6–3.8 times as long as the body diameter, with very thin walls, frequently poorly discernible, with proximal part short, tubular, and distal part large, swollen. Vagina extending inwards to 31–43% of body diameter, sigmoid. Vulva slightly protruding. Rectum short, 0.9–1.3 times the anal body width; three small gland-like cells are distinguishable around the intestine-rectum junction. Tail conical-elongate with acute terminus. Phasmids located at 34–40% of tail length from anus.

#### Male

Reproductive system monorchic, with testis reflexed ventrad anteriorly. Spicules paired and symmetrical: manubrium ventrally bent, angular hook-shaped, reduced calamus, and very curved ventrad lamina lacking dorsal hump, with well-developed ventral velum, narrower at its middle length, and spatulate tip in lateral view having a refringent forked axis. Gubernaculum well developed, slightly curved, about 0.4 times of the spicules length, with thin corpus. Three small gland-like cells are distinguishable around the beginning of the cloaca. Tail conical, slightly curved ventrad, conoid anterior to phasmid and them filiform. Genital papillae seven pairs, tree pre-cloacal (GP1 and GP2 subventral, GP3 lateral) and four post-cloacal: two pairs subventral (GP5, GP6) and one subdorsal (GP4) at the middle of tail length and one subdorsal (GP7) at beginning of the filiform part. One mid-ventral adcloacal papilla (MP) present. Phasmids at 32 to 50% of tail length from the cloacal aperture, close to genital papillae GP7.

### Remarks

The material examined in this study agrees well with the type population of *P. pycnus* described by Thorne (1938), especially in the morphology of the spicules although, unfortunately, Thorne (*op. cit.*) did not provide their measurements. Apparently, according to the drawing illustrations, the author did not observe the post-vulval uterine sac which is now know to be very large but (in the specimens examined in this study) its walls are very thin and sometimes not very well discernible. Also, the vagina structure was not illustrated with precision. Later, Hechler (1971a) described lectotype specimens of this species and observed the presence of post-vulval uterine sac but it was not described or illustrated. The main characters to identify this species, the spicules morphology (with manubrium having dorsal angular side and ventral hook-like side; Fig. 4) and morphometry (56–61 vs 54–70 µm, measured as a straight line, or chord, connecting the spicule manubrium with the lamina tip) agree perfectly with *P. pycnus*, while the gubernaculum is slightly longer (29–32 vs 25–27 µm). Unfortunately, most of the measurements were not provided in the previous records of the species (Table 2).

**Table 2.** Comparative morphometrics of *Panagrellus pycnus* Thorne, 1938.

Reference	Present paper		Thorne (1938)		Hechler (1971a)		Andrássy (2005)**	
Country	Italy		USA		USA		Hungary	
Sex	Female	Male	Female	Male	Female	Male	Female	Male
n	10	10	?	?	12	15	?	?
Body length (*L*)	993–1,258	931–1,322	1,000–1,400	800–1,200	1,170–1,410	900–1,222	1,170–1,410	800–1,200
a	23.6–29.6	24.6–29.9	18.0	21.0	16.0–21.5	20.6–26.9	16.0–22.0	21.0–27.0
b	5.3–16.5	4.6–6.5	8.0	6.8	6.1–8.0	5.7–7.4	6.1–8.0	5.7–7.4
c	6.8–8.1	7.8–10.9	8.5	9.0	7.6–10.4	7.9–10.4	8.0–10.0	8.0–10.0
c'	4.9–7.0	3.6–5.1	4.7*	4.2*	5.8*	4.7*	6.0	3.5–4.0
V	63–69	–	73	–	71–77	–	71–77	–
Stoma length	12–16	10–14	?	?	11–14	10–13	12–14	12–14
Spicules length (arc)	–	70–81	–	?	–	71–81*	–	?
Spicules length (chord)	–	56–61	–	?	–	54–70	–	50–70
Gubernaculum length	–	28–29	–	?	–	25–27	–	25–27

**Notes:** Measurements in μ m. *Measurement from drawings. **Measurements adapted from Hechler (1971). ? Unknown measurement. – Character absent.

On the other hand, the most similar species to *P. pycnus* is *P. leperisini* Massey, 1974, with which it could be confused by having similar spicules morphology. However, *P. pycnus* presents larger body (0.80–1.4 mm in Thorne’s description, 0.90–1.41 in Hechler’s description and 0.93–1.32 mm in the present paper vs 0.74–0.97 mm), lip region wider (wider than the adjacent part of body vs narrower) and longer spicules (70–81 vs 60 µm).

### Diagnosis

*Panagrellus pycnus* is characterized by having 0.93–1.32 mm long body, lip region continuous with the adjoining body, lips separated six having rounded sensilla, amphids small, stoma with gymnostom very reduced, pharynx with not swollen metacorpus, neck 161–203 µm long, excretory pore at level of the metacorpus, female reproductive system monodelphic-prodelphic with post-vulval uterine sac 99–162 µm long or 2.6–3.8 times as long as the body diameter divided in a short tubular proximal part and a long swollen distal part, vulva post-equatorial (*V* = 63–69), female tail conical elongate with acute terminus (133–170 µm, *c* = 6.8–8.1, *c′* = 4.9–7.0), male tail conical elongate with acute terminus (104–137 µm, *c* = 7.8–10.9, *c′* = 3.6–5.1), spicules 70–81 µm long having angular hook-shaped and very curved ventrad lamina ending in a spatulate tip with a refringent forked axis, and gubernaculum 29–32 µm long.

### Differential diagnosis

**Figure 5:**
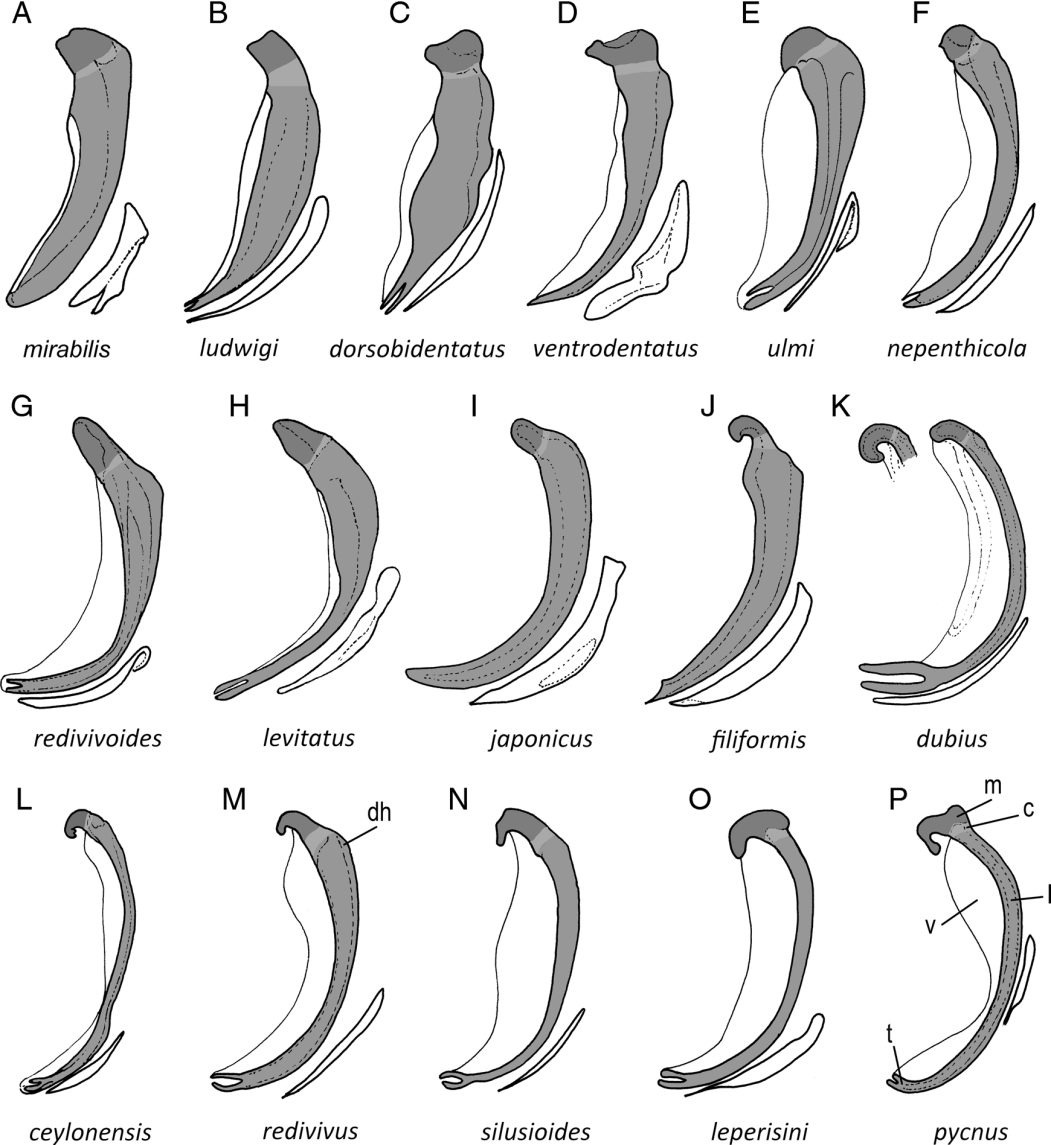
Morphology of the spicules and gubernacula of the species of the genera *Baujardia* Bert, Tandingan De Ley, Van Driessche, Segers and De Ley, 2003 and *Panagrellus* Thorne, 1938. (A) *Baujardia*; (B–D) *Panagrellus* species with truncated manubrium; (E–I) *Panagrellus* species with rounded or conoid manubrium; (E–P) *Panagrellus* species with curved or hook-shaped manubrium (c = calamus, dh = dorsal hump, l = lamina, m = manubrium, v = velum, t = tip).

The *Panagrellus* species are easily differentiated by the size (Table 3) and morphology (Fig. 5) of the spicules (Abolafia et al., 2016). Three main groups are distinguished according the morphology of the spicules. The first group, (Fig. 5B–D) with more simple spicules, includes three species [*P. dorsobidentatus* (Rühm, 1956) Baker, 1962, *P. ludwigi* (de Man, 1910) Goodey, 1945 and *P. ventrodentatus* (Heindl-Mengert, 1956) Baker, 1962] having robust spicules with irregular truncated manubrium, probably a plesiomorphic character. The second group (Fig. 5E–I), with five species [*P. japonicus* (Yokoo and Ota, 1961) Andrássy, 1984 *P. levitatus* *P. nepenthicola* (Menzel, 1922) Goodey, 1945, *P. redivivoides* (Goodey, 1943) Goodey, 1945 and, probably, *P. ulmi* Abolafia, Alizadeh and Khakver, 2016], presents ventrally curved spicules with rounded manubrium, straight or slightly ventral bent, and lamina with variable bifurcated tip. The third group (Fig. 5J–P), composed by seven species [*P. ceylonensis*, *P. dubius*, *P. filiformis* (Sukul, 1971) Andrássy, 1984 *P. leperisini*, *P. pycnus*, *P. redivivus* and *P. silusioides* Tsalolikhin, 1965], have ventrally curved spicules with ventral bent manubrium and lamina with well-developed bifurcated tip, probably an apomorphic character. Between this last group, the curvature degree of the manubrium is a diagnostic character. Thus, spicules with spirally curved manubrium appear in *P. ceylonensis* (with scarcely sigmoid lamina and short bifurcated terminus), *P. dubius* (with very curved lamina and long bifurcated terminus) and *P. filiformis* (with curved lamina while the terminus was not well illustrated). Manubrium poorly ventrally bent appears in *P. redivivus* and *P. silusioides*, having scarcely to very bent manubrium respectively, and slightly developed dorsal hump at lamina, while hook-shaped manubrium appears in some *P. ceylonensis* (narrow manubrium and poorly curved lamina), *P. leperisini* and *P. pycnus* (both species with wide manubrium and ‘C’-like lamina), more ventrally curved in *P. ceylonensis*, while *P. pycnus* is more anteriorly curved than *P. leperisini*.

**Table 3.** Length of the spicules (measured in arc) of the *Panagrellus* species with hooked manubrium.

References	Ceylonensis	Dubius	Filiformis	Leperisini	Pycnus	Redivivus	Redivivus as leucocephalus	Redivivus as parasiticus	Silusioides
Present paper	–	–	–	–	70–81	–	–	–	–
Goodey (1922)	–	–	–	–	–	70*	–	–	–
Steiner (1936)	–	–	–	–	–	–	?	–	–
Thorne (1938)	–	–	–	–	74***	–	–	–	–
Sandground (1939)	–	–	–	–	–	–	–	65–68*	–
Rühm (1956)	–	–	–	–	–	70–90* *ceylonensis*?	–	–	–
Tsalolikhin (1965)	–	–	–	–	–	–	–	–	75–90**
Sanwal (1960)	–	60–62*	–	–	–	–	–	–	–
Hechler (1970)	–	–	–	–	–	77*	–	–	–
Hechler (1971a)	–	56–60*	–	–	71–81*	64–74*	45*	–	–
Hechler (1971b)	81–89*	–	–	–	77–83*	–	–	–	–
Sukul (1971)	–	–	27*	–	–	–	–	–	–
Massey (1974)	–	–	–	60*	–	–	–	–	–
Stock and Nadler (2006)	83* *redivivus*? *silusioides*?	64* *dubius*? *leperisini*?	–	–	–	65* *ceylonensis*? *parasiticus*?	–	–	–

**Notes:** *Obtained from drawings. **Obtained from other measurements. ***Obtained from drawings in Goodey (1943). ?Presumable identity of the material examined by the author referenced.

### Molecular characterization and phylogenetic position

**Figure 6:**
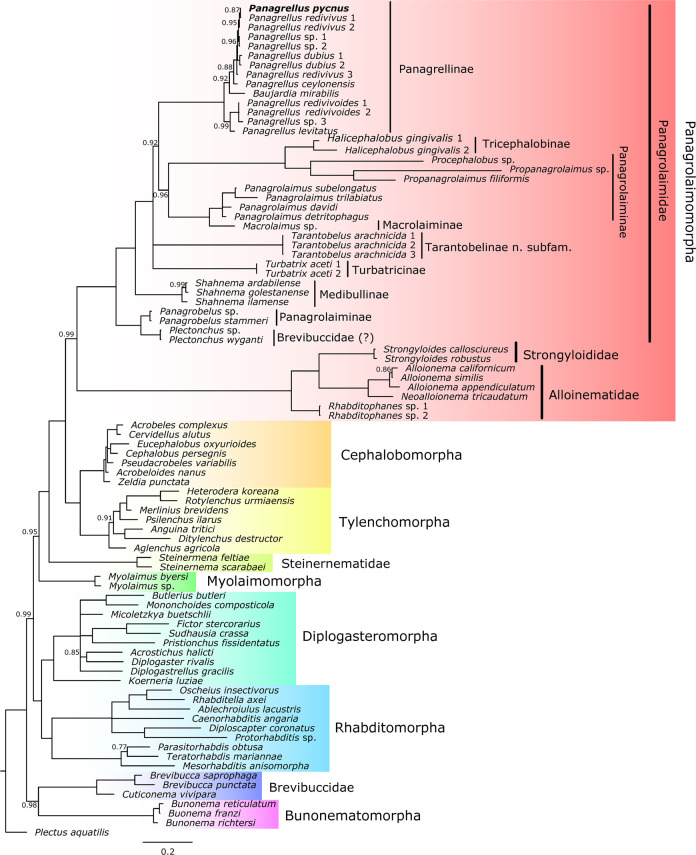
Bayesian Inference tree showing the phylogenetic position of *Panagrellus pycnus* and its related taxa based on a concatenated 18S and 28S rDNA regions. Bayesian posterior probabilities (pp) are given for each clade. Nodes with pp < 0.70 were collapsed. When pp = 1, its value is not shown. Scale bar shows the number of substitutions per site.

One 928 bp 18S rDNA sequence (GenBank accession number MZ656001) and one 766 bp 28S rDNA sequence (GenBank accession number MZ656000) were obtained for *P. pycnus*. After the molecular analysis, *P. pycnus* exhibits a high level of rDNA similarity with other *Panagrellus* species (Fig. 6).

## Discussion

### On the identity of *Panagrellus pycnus* and other related species

The morphology and morphometry of the material examined now agree with the type population of *Panagrellus pycnus* described by Thorne (1938) and redescribed by Hechler (1971a, b). The main characters to distinguish this material from other species are the size of the spicules (70–81 µm length and reaching the GP1) and the presence of a manubrium with angular dorsal side and hook-like ventral side. Two species, *P. ceylonensis* and *P. leperisini*, are similar to *P. pycnus*, but the former species differs in size (70–81 µm reaching the GP1 vs 81–89 µm length reaching the GP1 in *P. ceylonensis* and 56–62 µm length not reaching the GP1 in *P. leperisini*) and morphology (lamina ventrally more curved at first third *vs* homogeneously curved but ventrally convex at its mid-length in *P. ceylonensis* and slightly ventrally more curved anterior and posteriorly in *P. leperisini*).

Other species have very similar spicules and could be confused with *P. pycnus*. For example, some populations of *P. pycnus* could have been confused with *P. redivivus*. Thus, de Man (1914, as Anguillula silusiae), Goodey (1943), Hechler (1970, 1971a, Zullini (1982) and Ferris (2009) described several populations of *Panagrellus* agreeing with *P. redivivus*, showing specimens with spicules that do not reach the pre-cloacal GP1 and possessing a dorsal hump with a slight ventral bend to the calamus. However, the material examined by Corrêa de Carvalho and Álvares Corrêa (1953) and Rühm (1956) does not agree well with *R. redivivus* as observed Hechler (1971a); thus, the material described by Corrêa de Carvalho and Álvares Corrêa (op. cit.) shows spicules reaching the GP1 (*vs* not reaching the GP1 in *P. redivivus*, see de Man, 1914 as *P. silusiae* (Aubertot, 1925; Goodey, 1922; Hechler, 1971a) having almost angular hook-like manubrium; on the other hand, the material described by Rühm (op. cit.) is very similar to *P. ceylonensis* agreeing in measurements, excretory pore at basal bulb level and spicules slightly sigmoid with narrow hooked manubrium.

On the other hand, Stock and Nadler (2006) characterized morphological and molecularly three species of the genus *Panagrellus*. However, there are some discrepancies about their identity. With respect to the morphology of the illustrated spicules, the spicule named as ‘*P. ceylonensis*’ (see Fig. 3B) is more similar to *P. redivivus* and *P. silusioides* by having ventral bent manubrium and wide velum; the spicule named as ‘*P. redivivus*’ (see Fig. 3D) agrees with *P. ceylonensis* (although it is unusually short, 65 µm long) and *P. parasiticus* (junior synonym of *P. redivivus*) by having spirally curved manubrium and narrow velum; and the spicule named as ‘*P. dubius*’ (see Fig. 3E), agreeing with this species by having long bifurcated terminus but also it is similar to *P. leperisini* by the size and by having angular hook-like manubrium. However, other morphological and morphometrical characters are necessary to correctly identify these species.

### On the phylogenetic position of *Panagrellus pycnus* in the genus *Panagrellus*

The material examined now of *Panagrellus pycnus* is phylogenetically related with other sequenced species of the genus *Panagrellus*. The species included in this genus are characterized by having very short gymnostom with minute rhabdia, post-vulval uterine sac differentiated in a short tubular proximal part and a large swollen distal part, and female and male tails conoid-elongate. An important character of the spicules is the ventral curvature and hook-shaped manubrium. There are two primary clades in the rDNA phylogenetic tree of *Panagrellus* (Fig. 6). One includes the species *P. levitatus* and *P. redivivoides* and is characterized by the more robust spicules with rounded manubrium, like other genera of the family Panagrolaimidae. The second clade includes *P. ceylonensis*, *P. dubius*, *P. pycnus* and *P. redivivus* and is characterized by thinner and elongate spicules, increasing the curvature of the manubrium from slightly ventrally curved to hook-like.

### Phylogenetic position of the genus *Panagrellus* and its related genera

*Panagrellus* is recovered as paraphyletic (Fig. 6) with *Baujardia* Bert, Tandingan De Ley, Van Driessche, Segers and De Ley, 2003 located inside the genus. Indeed, *Baujardia* is very similar to *Panagrellus*, only differentiated from it by the morphology of the stoma, slightly longer in *Baujardia*. Unfortunately, only a sequence based on 18S rDNA was obtained of *Baujardia mirabilis* Bert, Tandingan De Ley, Van Driessche, Segers and De Ley, 2003, the only species of the genus, leading to the possible consideration of the synonymy of *Baujardia*.

With respect to the subfamily Panagrellinae Andrássy, 1976 containing the genera *Baujardia* and *Panagrellus*, is shown as monophyletic. This subfamily was synonymized with Baujardinae Andrássy, 2005 by Abolafia et al. (2016) and the present molecular analysis agree with these authors.

Other subfamilies of the family Panagrolaimidae as Medibullinae Siddiqi, 1993 [tranferred to Panagrolaimidae by Abolafia and Peña-Santiago (2018), including *Shahnema* Siddiqi, 2014] Tricephalobinae Andrássy, 1976 (including *Halicephalobus* Timm, 1956 and Turbatricinae Goodey, 1943 (including *Turbatrix* Peters, 1927) are shown as monophyletic.

Conversely, the subfamily Panagrolaiminae is shown as polyphyletic. Four genera belonging to this subfamily, *Panagrobelus* Thorne, 1939, *Panagrolaimus* Fuchs, 1930 *Procephalobus* Steiner, 1934 and *Propanagrolaimus* Andrássy, 2005 appear not directly related, unlike what was proposed by Andrássy (1984, 2005). However, additional morphological and molecular studies are necessary to confirm the relationships between these genera and, after that, it could be suitable to reestablish the subfamily Procephalobinae Paramonov, 1956 to include *Procephalobus* and *Propanagrolaimus*, among others.

Nevertheless, the genus *Macrolaimus* Maupas, 1900 currently belonging to the family Chambersiellidae Thorne, 1937 subfamily Macrolaiminae Sanwal, 1971, appears related with *Panagrolaimus*. Unfortunately, not enough species have been sequenced to clarify its phylogenetic position. To this respect, Kitagami et al. (2019) sequenced a species identified as *Panagrolaimus* sp. (LC382049), however, it agrees with some species of the genus *Macrolaimus* according the stoma morphology being showed in the present phylogenetic tree belonging to this last genus (see Supplementary Fig. S1 in Kitagami et al., op. cit.).

On the other hand, the genera *Plectonchus* Fuchs, 1930 and *Tarantobelus* Abolafia and Peña-Santiago, 2018 proposed as belonging to the family Brevibuccidae Paramonov, 1956 by Andrássy (1976, 1984, 2005) and Abolafia and Peña-Santiago (2018), respectively, appear not closely related with this family in this new study. According to this, both genera are transferred to the family Panagrolaimidae. Regarding the genus *Plectonchus* (with post-equatorial vulva and without post-vulval uterine sac) appears related to the panagrolaimid genus *Panagrobelus* (with equatorial vulva and very short post-vulval uterine sac); however with only limited 18S rDNA sequence in support of the genus, there is a clear need to obtain detailed morphological and molecular data, without which the genus should be considered *incertae sedis* until to obtain a detailed morphological-molecular study of its species. Secondly, the genus *Tarantobelus* appears forming part of a well-supported, separate clade which is proposed now as a new subfamily, Tarantobelinae n. subfam., clearly differentiated from other subfamilies, especially by having short and robust pharyngeal isthmus and vulva post-equatorial. This new subfamily is composed by the only genus *Tarantobelus*.

## Taxonomic account

Tarantobelinae n. subfam.

Diagnosis: small body with cuticle nearly smooth, lateral field inconspicuous, lip region with six separated lips, each bearing a small cuticular flap topping it, panagrolaimoid stoma with well-developed gymnostom having broad and refringent rhabdia, panagrolaimoid pharynx with isthmus robust and slightly longer than basal bulb, excretory pore situated at level of isthmus, female reproductive system monodelphic-prodelphic, length of post-vulval sac less than the corresponding body diameter, vulva post-equatorial and distinctly protruding, female tail conical with acute tip, male tail conical with a long and thin mucro, spicules curved ventrad with rounded manubrium, and thick gubernaculum.

**Table S1.** GenBank accession numbers of the species used in the phylogenetic tree.

Species	18S rDNA	28S rDNA
*Ablechroiulus lacustris*	EU196013	EU195976
*Acrobeles complexus*	KU180671	DQ145620
*Acrobeloides nanus*	DQ102707	DQ903076
*Acrostichus halicti*	HQ130163	HQ130212
*Aglenchus agricola*	KJ869356	KP835679
*Alloionema appendiculatum*	FJ665982	KP204846
*Alloionema californicum*	KX017492	NA
*Alloionema similis*	KX185606	NA
*Anguina tritici*	AY593913	KC818620
*Baujardia mirabilis*	AF547385	NA
*Brevibucca punctata*	NA	DQ077787
*Brevibucca saprophaga*	EU196018	KU180677
*Bunonema reticulatum*	EU196017	EU195989
*Bunonema richtersi*	FJ040451	NA
*Buonema franzi*	AJ966477	NA
*Butlerius butleri*	KP453998	NA
*Caenorhabditis angaria*	JN636068	JN636068
*Cephalobus persegnis*	AY284663	AF143368
*Cervidellus alutus*	AF202152	KU180683
*Cuticonema vivipara*	EU196019	EU195991
*Diplogaster rivalis*	KJ636326	NA
*Diplogastrellus gracilis*	KJ877216	KJ877249
*Diploscapter coronatus*	AY593921	NA
*Ditylenchus destructor*	KJ636422	MN307126
*Eucephalobus oxyurioides*	AY284665	HM439768
*Fictor stercorarius*	KJ877235	KJ877282
*Halicephalobus gingivalis* 1	JX674039	JX194163
*Halicephalobus gingivalis* 2	NA	KU180686
*Heterodera koreana*	MZ027493	MZ027488
*Koerneria luziae*	AB597232	KJ877284
*Macrolaimus* sp.	LC382049	NA
*Merlinius brevidens*	KX789708	NA
*Mesorhabditis anisomorpha*	AF083013	NA
*Micoletzkya buetschlii*	JX163973	NA
*Mononchoides composticola*	KP067833	NA
*Myolaimus byersi*	KU180665	KU180676
*Myolaimus* sp.	NA	DQ145643
*Neoalloionema tricaudatum*	KR817916	KR817917
*Oscheius insectivorus*	AF083019	EU195968
*Panagrellus ceylonensis*	NA	DQ408251
*Panagrellus dubius* 1	NA	DQ145648
*Panagrellus dubius* 2	NA	DQ408252
*Panagrellus levitatus*	KY126845	NA
*Panagrellus pycnus*	MZ656001	MZ656000
*Panagrellus redivivoides* 1	MH608262	MH608297
*Panagrellus redivivoides* 2	MH608263	MH608298
*Panagrellus redivivus* 1	AF083007	DQ408250
*Panagrellus redivivus* 2	MK541674	MK541658
*Panagrellus redivivus* 3	NA	AF331910
*Panagrellus* sp. 1	MN082326	NA
*Panagrellus* sp. 2	MH608264	MH608299
*Panagrellus* sp. 3	KP876562	KM489128
*Panagrobelus sp.*	LC382079	NA
*Panagrobelus stammeri* 1	FJ969134	NA
*Panagrolaimus davidi*	AJ567385	AY878385
*Panagrolaimus detritophagus*	EU543176	GU014547
*Panagrolaimus subelongatus*	KY119431	NA
*Panagrolaimus trilabiatus*	KF011487	NA
*Parasitorhabdis obtusa*	EU003189	EF990724
*Plectonchus* sp.	AY593920	NA
*Plectonchus wyganti*	KJ636307	NA
*Plectus aquatilis*	AY284700	EF417147
*Pristionchus fissidentatus*	KJ877237	KJ877273
*Procephalobus* sp.	EU543179	NA
*Propanagrolaimus filiformis*	KJ636392	NA
*Propanagrolaimus* sp.	KJ434175	NA
*Protorhabditis* sp.	AF083001	AY602168
*Pseudacrobeles variabilis*	AF202150	NA
*Psilenchus ilarus*	MK639403	MW716284
*Rhabditella axei*	NA	AY602177
*Rhabditophanes* sp. 1	JX674037	JX674036
*Rhabditophanes* sp. 2	AF202151	AY294185
*Rotylenchus urmiaensis*	KP718970	KP718967
*Shahnema ardabilense*	KM454872	KM454873
*Shahnema golestanense*	KM454874	KM454875
*Shahnema ilamense*	KM454870	KM454871
*Steinermena feltiae*	FJ040419	NA
*Steinernema scarabaei*	FJ040424	AY172023
*Strongyloides callosciureus*	AB272231	AB272231
*Strongyloides robustus*	AB272232	NA
*Sudhausia crassa*	KJ877232	KJ877279
*Tarantobelus arachnicida* 1	MG669658	MF177710
*Tarantobelus arachnicida* 2	MZ655998	MZ656002
*Tarantobelus arachnicida* 3	MZ655999	MZ656003
*Teratorhabdis mariannae*	EF990716	EF990721
*Turbatrix aceti* 1	AF202165	AY294184
*Turbatrix aceti* 2	KU180673	KU180690
*Zeldia punctata*	NA	DQ145662
